# Sequential model based on human cognitive processing to robot acceptance

**DOI:** 10.3389/frobt.2024.1362044

**Published:** 2024-03-15

**Authors:** Waka Saeki, Yoshiyuki Ueda

**Affiliations:** ^1^ Graduate School of Education, Kyoto University, Kyoto, Japan; ^2^ Institute for the Future of Human Society, Kyoto University, Kyoto, Japan

**Keywords:** robot acceptance, human-robot interaction, appearance, politeness, human cognitive process

## Abstract

Robots have tremendous potential, and have recently been introduced not only for simple operations in factories, but also in workplaces where customer service communication is required. However, communication robots have not always been accepted. This study proposes a three-stage (first contact, interaction, and decision) model for robot acceptance based on the human cognitive process flow to design preferred robots and clarifies the elements of the robot and the processes that affect robot acceptance decision-making. Unlike previous robot acceptance models, the current model focuses on a sequential account of how people decide to accept, considering the interaction (or carry-over) effect between impressions established at each stage. According to the model, this study conducted a scenario-based experiment focusing on the impression of the first contact (a robot’s appearance) and that formed during the interaction with robot (politeness of its conversation and behavior) on robot acceptance in both successful and slightly failed situations. The better the appearance of the robot and the more polite its behavior, the greater the acceptance rate. Importantly, there was no interaction between these two factors. The results indicating that the impressions of the first contact and interaction are additively processed suggest that we should accumulate findings that improving the appearance of the robot and making its communication behavior more human-like in politeness will lead to a more acceptable robot design.

## 1 Introduction

The increasing labor shortage in recent years has facilitated the need to replace human activities with robots. Especially, the number of people in their over 50 s will be increased in OECD countries in the future (Acemoglu and Restrepo, 2017). Acemoglu and Restrepo (2017) argue that the use of robots and AI can help compensate for a declining working population. Examples include the use of machine-type robots in factory production lines (e.g., assembling engines on an automobile production line) and humanoid robots in situations that require communication with others, such as hotel receptionists and meal servers (e.g., providing information on the hotel’s facilities and services, greeting the customers, and delivering food to them). The use of robots not only addresses the issue of labor shortages but also improves the quality of life. For example, when assisting in the care of elderly people, communication with robots improving their ability to perform activities of daily living (ADL) (e.g., PaPeRo i robot produced by the NEC Corporation; Mizuno et al., 2021). Some robots can mitigate loneliness and become intimate buddies (e.g., Vector produced by Anki; Odekerken-Schröder et al., 2020). Furthermore, the use of robots in educational settings is expected to help students develop academic skills and engage in learning experiences (for a review, Toh et al., 2016).

Robots can help people, but before that, robots must be introduced to the field. However, there are several barriers to the introduction of these robots. For example, organizations do not accurately understand or perceive the costs and benefits of using robots (Mutlu and Forlizzi, 2008). In addition, robots are not always well accepted by users, and their mere presence sometimes leads to negative impressions (i.e., Mori, 1970; Nomura et al., 2004; Urakami and Sutthithatip, 2021). If robots are expected to play a helping role, it is important to consider how to encourage people to accept them. In this study, we propose a model based on sequential human cognitive processing for decision-making regarding robot acceptance (or rejection) and empirically verify its structure.

One of the well-known models is the technology acceptance model (TAM), proposed by Davis (1985,1989). This model describes people’s attitudes toward accepting the use of information systems. TAM incorporates the perceived usefulness and ease of use of technology as a predictor, with a coefficient of determination of 0.3–0.4 for people’s choice to accept or reject IT (Holden and Karsh, 2010). Heerink et al. (2010) extended the TAM by adding variables related to social interaction; more recently, Wirtz et al. (2018) further added other theoretically relevant concepts and summarized them as the service robot acceptance model (sRAM). The pivotal point of sRAM is that customer acceptance of service robots is supported not only by the functional elements proposed in the TAM but also by social-emotional elements (e.g., humanness and interactivity) and relational elements (e.g., trust and rapport). The approach of adding social factors to the TAM to explain robot acceptance has also been applied in other acceptance models (e.g., the persuasive robot acceptance model [PRAM]; Ghazali et al., 2020; and the robot acceptance model for care or RAM care; Turja et al., 2020). Although these models indicates that social factors such as social-emotional elements and relational elements are necessary for evaluating robot acceptance, they are not necessarily separate in robot design. For example, the more a robot looks like a human (i.e., perceived humanness), the more likely people are to sympathize with it and perceive it as warm, competent, and less uncomfortable (i.e., positively evaluated in relational elements) (Riek et al., 2009; Stroessner and Jonathan, 2019). Thus, the impressions of a robot (considered relative elements) may vary as a function of its human-likeness (Prakash and Rogers, 2015).

Furthermore, we should consider that these elements are not simultaneously provided but are processed sequentially. This is a reasonable assumption given that in most cases, “seeing” the robot occurs prior to interacting with it. Therefore, the perception of the robot’s appearance causes the impression of the robot, which is a prerequisite for interacting with the robot. Perceived usefulness, as conceptualized in the TAM, does not exist in isolation but is always formed by impressions obtained through a combination of such appearances and interactions. Therefore, in this study, the elements for robot acceptance were decomposed and reconstructed based on sequential processing stages. The relationships between each of the processing stages were subsequently examined via a scenario-based experiment.

**FIGURE 1:**
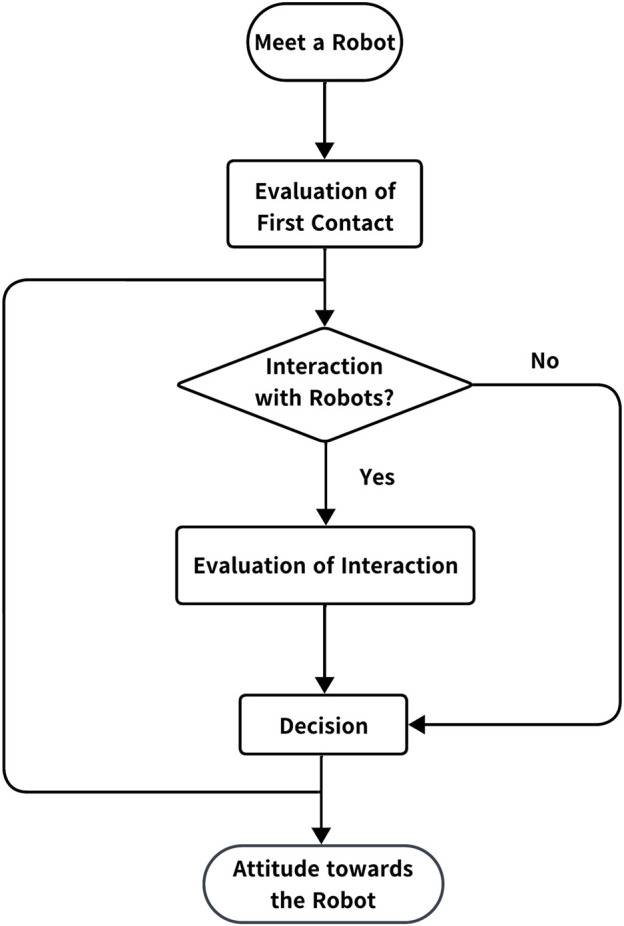
Robot acceptance model.

To determine whether a person can accept robots, they are expected to undergo three processing stages—first contact, interaction, and decision. In their first contact, people obtain an impression of the robot at a glance based on its appearance (e.g., Prakash and Rogers, 2015; Riek et al., 2009; Stroessner and Benitez, 2019). In the interaction stage, people update and establish an impression of the robot. Edwards et al. (2019) demonstrated that participants have a more favorable impression of a robot when interaction emulates human–human communication. Furthermore, robots using hedges (i.e., “I think,” “probably”) or discourse markers (i.e., “I mean,” “so”) in their communication receive more favorable evaluations than when they are not used (Torrey et al., 2013). In the decision stage, people decide whether the robot is acceptable or unacceptable based on the impressions received during the first contact and interaction stages. Figure 1 shows the proposed impression-processing model for robot acceptance. Each element follows those proposed in previous studies, but we arranged them to follow the flow of human cognitive processing: The impressions that people have in the first contact stage are strengthened or changed according to the action and validity of the response, as well as the comfort they feel when interacting with it. The decisions regarding the acceptability of the robot were made based on these impressions. It should be noted that the first contact is not always followed by interaction, and decisions are sometimes made immediately after the first contact. Furthermore, once a decision is made, it may not necessarily be permanent, and subsequent interactions are likely to successively update the decision to accept the robot.

Previous studies have focused on robots that provide good impressions in either the first contact or interaction stage (Riek et al., 2009; Stroessner and Benitez, 2019; Torrey et al., 2013; Prakash and Rogers, 2015; Edwards et al., 2019). However, considering that such sequential cognitive processing is involved in the decision of robot acceptance, it is necessary not only to investigate impressions at each stage independently (i.e., assuming an additive system) but also to examine whether the effect of interaction impressions differs depending on the impression of the first contact (i.e., interaction relationships between the stages). This study hypothesizes that if the robot behaves in the same way, but the evaluation of acceptance for it varies with its appearance, then the interaction between appearance and politeness on the decision of robot acceptance will be significant.

In this study, we conducted a scenario-based experiment, which helped individuals independently manipulate the impressions of their first contact and performance during the interaction. These scenario methods are effective in various fields (Wright et al., 2003) and are often used in human–robot and human–computer interaction studies (e.g., Friedman, 1995; Leo and Huh, 2020; Furlough et al., 2021). In each scenario, the robots were presented with pictures of a variety of appearances that gave different impressions, and they succeeded or slightly failed in their tasks in a service situation. Among the various actions of robots, this study primarily focuses on politeness behaviors, which refer to how service agents are seen as thoughtful and reliable (Brown and Swartz, 1989) and have a significant impact on trust (Coulter and Coulter, 2002). For example, when a robot initiated a conversation with a greeting, the participants perceived the robot to possess greater politeness than when it did not, and the impolite robot perceived heightened levels of intimidation, diminished equity, and reduced affability (Inbar and Meyer, 2019). Moreover, politeness encourages people to repeatedly interact with robots, even if they make minor mistakes (Saeki and Ueda, 2024). If the effect of politeness on the evaluation of robots differs according to the appearance of the robot, the interaction effect between the robot’s impression of appearance and politeness on their evaluation should be observed.

## 2 Experiment

The experiment was approved by the Institutional Review Board of the Graduate School of Education, Kyoto University (CPE-572).

In the experiment, three levels of robot appearance (high, medium, and low) were presented with four different interaction patterns (polite or casual robot behavior × success or minor failure in task), so there were 12 scenario patterns in total. Because the decision criteria may differ between when a task goal was achieved and when minor failures occurred, as indicated by prior studies (e.g., Inbar and Meyer, 2019; Leo and Huh, 2020; Saeki and Ueda, 2024), the analysis was conducted separately for task success and minor failure scenarios (6 scenario patterns for each) before beginning the analysis (i.e., planned comparison).

### 2.1 Materials and methods

#### 2.1.1 Participants

We recruited 80 participants (mean age = 41.0, *SD* = 10.1, range = 21–63; 41 males and 39 females) using a crowdsourcing site (Lancers, http://www.lancers.jp). All participants provided informed consent before participating in the experiment. The sample size was calculated using PANGEA (v0.2) (Westfall, 2016) with a within-participant factorial design, appearance (high vs. medium vs. low) × politeness (polite vs. casual), a medium effect size of.25 (Cohen, 1998), and an appropriate sample size to ensure power = 80 at a significance level of 5%. The required sample size for the interaction between the two factors was 54. Considering that some people may have to be excluded owing to the attention check and based on the outcomes of our previous study (Saeki and Ueda, 2024), we decided to obtain 80 samples in this study before starting data collection. Given that four participants had incomplete data, we analyzed the data from 76 participants (mean age = 42.1 years, *SD* = 10.1 years, range = 21–63 years; 37 males and 39 females). These participants also passed an attention check, which required them to read the text carefully and answer it correctly as instructed (see Gawronski et al., 2017 for the original version).

#### 2.1.2 Stimuli

The stimuli presented to the participants consisted of a scenario and robot pictures.

**Scenario.** The four scenarios were variations (politeness: polite vs. casual; success of the task: success vs. minor failure) of one basic scenario (service scene). The basic scenario was chosen from those used in our previous study (Saeki and Ueda, 2024). However, we replaced “an android-type robot” with “a humanoid-type robot” and inserted “as shown in the above image” according to the situation of this study. An example of a scenario (polite and successful) is presented below:

You have come to a department store to purchase a birthday present. This department store has a humanoid-type robot, as shown in the above image, installed at the reception desk. You ask at the reception where you could purchase a leather bag. The reception robot looks at you and bows while saying, “Welcome to our department store. How may I help you, sir (or ma’am)?” Then, the robot approaches you and explains with a gesture, saying, “If you would like to browse leather bags, you should go to Shop A. If you proceed straight down that aisle, turn right at the end, and go straight for a while, you will find Shop A on your right,” pointing in the direction of the destination. And, the robot adds “I hope you are able to find a good bag.” You proceed straight in the direction shown by the robot, turn right at the end, and go straight.

You successfully find Shop A on your right.

For other scenarios, see the appendix of Saeki and Ueda (2024). All the scenarios were presented in Japanese. For each scenario, the following questions were presented: “Would you like to interact with this robot again (= future interaction motivation),” “How much do you think this robot helped you accomplish the task (= contribution to achieving a goal)” with a 7-point Likert scale (1 = strongly disagree, 7 = strongly agree).

**Robot picture.** Twelve robot pictures, four each with high, medium, and low valences, were used in this study. These pictures were selected from the Anthropomorphic roBOT (ABOT) Database (Phillips et al., 2018) based on a preliminary evaluation experiment.

**FIGURE 2:**
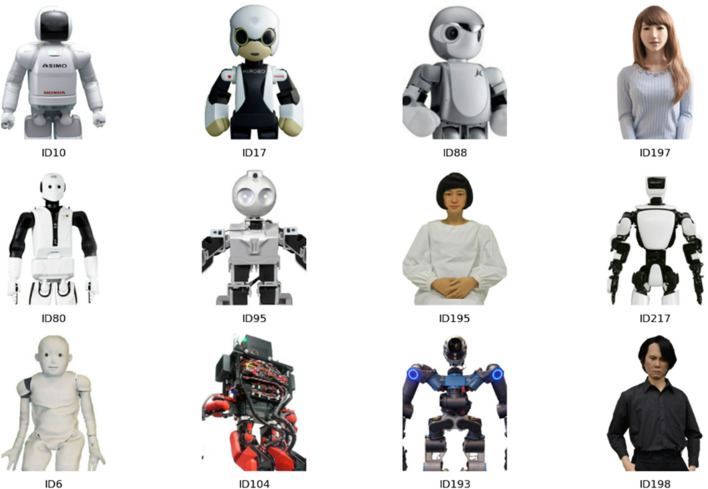
Robot stimulus. The top, middle, and bottom columns show the robots with high, middle, and low appearance impressions, respectively.

In the preliminary evaluation experiment, 69 sufficient-resolution images of robots with heads, torsos, hands, and legs (i.e., humanoid robots) were selected from the database and presented to 30 participants (mean age = 42.1 years, *SD* = 7.3 years, range = 30–63; 24 males and 6 females) in Lancers; these participants were different from those who participated in the main experiment. The image sizes were standardized to 400 and 300 pixels. The participants evaluated the familiarity, eeriness, likability, trustworthiness, dominance, extroversion, and attractiveness of each stimulus using a 7-point Likert scale (1 = strongly disagree, 7 = strongly agree). This evaluation can be regarded as the impression of the first contact based on the robot’s appearance. We conducted a principal component analysis on these evaluations and found that the first principal component (PC1) loaded strongly on familiarity and trustworthiness, suggesting a valence evaluation. This factor accounted for 78.3% of the variance in the evaluations. The second principal component (PC2) loaded strongly on eerie and extroversion, suggesting that a dominance evaluation accounted for 12.1% of the variance. Here, PC1 and PC2 were identical to those used in previous studies (Oosterhof and Todorov, 2008; Ueda et al., 2019). Twelve robots, four each with high, medium, and low valences, were selected based on the scores of PC1 to equalize the scores of PC2. The selected robot IDs in the ABOT database were 10, 17, 88, and 197 for high appearance impression (the mean PC1 score was 4.18 [ranged 3.32–5.12]); 80, 95, 195, and 217 for medium appearance impression (the mean PC1 score was 0.27 [ranged 0.00–0.58]); and 6, 104, 193, and 198 for low appearance impression (the mean PC1 score was −3.70 [ranged −4.23–2.93]). Figure 2 shows the robot stimuli used in this study.

#### 2.1.3 Procedure

The participants accessed a webpage created by Qualtrics via their computers or tablets to participate in the experiment. After signing the informed consent form, a scenario and a robot picture were presented, and the participants evaluated the robot agent (for detailed instructions, see Saeki and Ueda, 2024). Four scenarios were presented six times each with pictures of high-, medium-, and low-impression robots (twice for each type of robot). Robot images were randomly chosen for each trial. Therefore, 24 trials were performed, and the experiment was conducted at each participant’s own pace.

After 24 trials, the participants were sequentially presented with all the robot pictures used in this study, and their preference was evaluated using a 7-point Likert scale (1 = strongly dislike it, 7 = strongly like it) to check the validity of the robot picture impressions.

Once the participants answered each robot’s preference, they underwent an attention check, which was similar to the previous scenario study (Gawronski et al., 2017). Finally, the participants answered questions concerning their age and sex.

#### 2.1.4 Analysis

The dependent variables were the attitude toward robot acceptance, which were measured with the answers to two questions. The independent variable is the impression of the first contact (manipulated by robot appearances) and the impression of the interaction (manipulated by robot polite behaviors).

All analyses were performed using Python version 3.9.1 and R 4.2.3. In success or minor failure situations, we separately performed a 3 (appearance: high vs. medium vs. low) × 2 (politeness: politeness vs. casual) ANOVA on the evaluations concerning future interaction motivations and contribution to achieving a goal.

## 3 Results

### 3.1 Manipulation check

Among the preference ratings, the mean values were calculated for the high, medium, and low groups (for the high, medium, and low groups, the mean and SD were 4.10 and 0.86, 2.94 and 0.85, and 1.81 and 0.77, respectively), and a one-way repeated-measures ANOVA was conducted. There was a significant effect of impression, *F* (2, 150) = 330.92, *p* < .001, η_p_^2^ = .82. Multiple comparisons revealed significant differences between the impression robots (high > medium > low, *p*s < 0.001), indicating that the manipulation of the robot impressions according to the preliminary evaluation experiment was valid for participants in the real experiment.

### 3.2 Success situations

**FIGURE 3:**
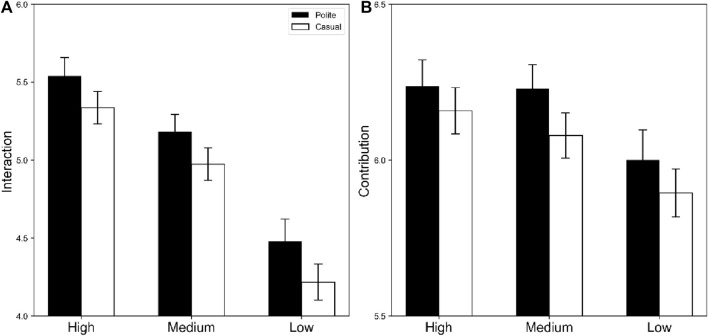
Means and standard errors of evaluations for robots in success scenarios. Black and white bars indicate polite and casual situations, respectively. Panels **(A)** display future interaction motivation; and **(B)** indicate contribution to achieving a goal.

Figure 3 shows the evaluations of the robots in the success scenarios.

**Future Interaction Motivation.** There was a significant main effect of appearance, *F* (2, 150) = 53.39, *p* < .001, η_p_^2^ = 42. Multiple comparisons revealed significant differences among the robot appearances (high > medium > low, *p*s < 0.001). The results showed that participants desired to interact with the high-, medium-, and low-appearance robots again, in that order. The main effect of politeness was also significant, *F* (1, 75) = 6.16, *p* = 0153, η_p_^2^ = .08, showing that more polite robots were desirable for interaction with each other than casual robots were. The interaction between impressions and politeness was not significant, *F* (2, 150) = 0.04, *p* = 961, η_p_^2^ = .0005, suggesting that future interaction motivations depending on robot politeness did not change according to robot appearance.

**Contribution.** There was a significant main effect of appearance, *F* (2, 150) = 5.42, *p* = .0053, η_p_^2^ = .07. Multiple comparisons revealed significant differences in high and low appearances (High > Low, *p* = .005) and in medium and low appearances (Medium > Low, *p* = .008), revealing that high- and medium-preference robots were perceived as contributing more than low-preference robots were. There was a marginal tendency indicating that polite robots tended to be perceived as more contributing than casual robots, *F* (1, 75) = 3.6, *p* = 0616, η_p_^2^ = 05. The interaction between appearances and politeness was not significant, *F* (2, 150) = 0.10, *p* = 902, η_p_^2^ = 0014.

### 3.3 Minor failure situations

**FIGURE 4:**
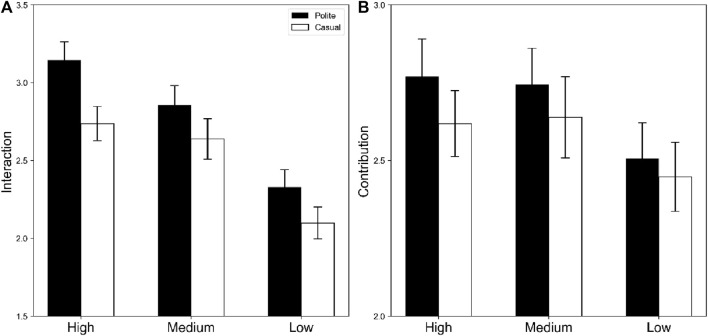
Means and standard errors of evaluations for robots in minor failure scenarios. The black and white bars indicate polite and casual situations, respectively. Panels **(A)** display future interaction motivation; and **(B)** indicate contribution to achieving a goal.

Figure 4 shows the evaluations of agents in minor failure scenarios.

**Future Interaction Motivation.** There was a significant main effect of appearance, *F* (2, 150) = 21.35, *p* < .001, η_p_^2^ = 22. Multiple comparisons revealed significant differences in high and low appearances (High > Low, *p* < 0.001) and in medium and low appearances (Medium > Low, *p* < 0.001), revealing that high- and medium-preferable robots were more likely to interact than low-preference robots were, even when they performed a slight failure in their task. The main effect of politeness was also significant, *F* (1, 75) = 8.47, *p* = 0048, η_p_^2^ = 01, showing that more polite robots were desirable for interaction with each other than casual robots were. The interaction between appearances and politeness was not significant, *F* (2, 150) = 0.50, *p* = .610, η_p_^2^ = 0066.

**Contribution.** There were no significant main effects of appearance or politeness or their interactions (*F*s < 2.49, *p*s >.05). These factors did not affect the contribution to achieving a goal when the robot agent failed slightly during the task.

### 3.4 Exploratory analysis

We examined the correlation of ratings between the future interaction motivation and contribution by averaging the factors of appearance and politeness, and found a moderate correlation of *r* = 48 in success situations and a large correlation of *r* = 72 in minor failure situations.

## 4 Discussion

In this study, we decomposed and reconstructed the robot acceptance model based on sequential processing stages with elements proposed in previous studies (e.g., Riek et al., 2009; Stroessner and Benitez, 2019; Torrey et al., 2013; Prakash and Rogers, 2015; Edwards et al., 2019). Throughout the experiment, we provided a sequential account of how individuals make decisions about accepting a communication robot from the moment they encounter it and how the information processing at each stage is interconnected. The outcomes of the scenario-based experiment showed that when the task was successful, the high-, medium-, and low-preference robots, in that order, desired to interact again. Moreover, robots that behave politely are also desirable for interaction again. High- and medium-preferable robots were perceived as contributing more than low-preferable robots were. When the task was a minor failure, high- and medium-preferable robots and polite robots were more likely to interact again than low-preferable robots and casual robots, respectively. However, there was no significant effect of appearance or politeness on the perception of contributions. Interestingly, in both situations, there was no significant interaction effect between appearance and interaction impressions, suggesting that appearance and interaction impressions had separate impacts on the decision to accept robots.

In our previous study, wherein the same scenarios were used (Saeki and Ueda, 2024), the impact of politeness could be observed in the robot’s contribution judgment and future interaction motivation. However, in this study, the effect of politeness was observed only for future interaction motivation. This difference may be attributed to the difference in the robot type. Saeki and Ueda (2024) presented androids with a head, torso, two arms, two legs, and a physical appearance similar to that of humans; however, in this study, androids and humanoids, which are less human-like, were presented. Because similarity to humans causes anthropomorphism and people generally tend to have positive impressions (e.g., Duffy, 2003; Lu et al., 2021), participants in this study were likely to have less anthropomorphism, weakening the effect of politeness than in human–human and human–android interaction situations.

The results showed no interaction between impressions of the first contact and interaction on robot acceptance, suggesting that the impressions formed in each stage are additively processed. This means that the appearance and behavior of robots can be treated as independent modules, which supports the findings of previous studies that investigated each of them separately. At least in the setting of this study, robots with a good appearance tend to be accepted even if their behavior is a little strange (i.e., minor task failure), and robots that behave politely also tend to be accepted even if their appearance is not necessarily favorable. However, it is important to consider whether this approach can be adapted to the behavior of every type of robot. In general, when humans and robots have the same interactions, they are rated in approximately the same manner (Inbar and Meyer, 2019). Moreover, people with trustworthy facial impressions are more likely to be perceived as trustworthy in their behaviors (Yu et al., 2014). Although the assumption that impressions of first contact and interaction with a robot are additives in robots that demand politeness, such as reception desks, meal services, and security guards, seems reasonable, further investigation is necessary for situations that require other characteristics in interaction (e.g., more intimacy in care situations for the elderly (Odekerken-Schröder et al., 2020; Mizuno et al., 2021)).

Thus, the three-stage processing model for robot acceptance proposed in this study is beneficial for examining how each element of a robot affects human decision-making and how the robot should be designed. This model allows us to estimate which factors play an important role in the acceptance of a specific robot.

This study focused on politeness as a specific example of interaction and was limited to service situations. Those are because politeness is important in human-human and human-robot communication, and service situations would be easy for participants to imagine. However, robots have recently been introduced into situations that require a high level of expertise other than simple service situations that are relatively easy for anyone to perform, and the type of these services can also affect robot acceptance (Xiao and Kumar, 2019). The model proposed in this study is the first step toward a robot acceptance model based on human cognitive flow, but the possibility of adapting the model to these situations is still under way. Based on these findings, future research should expand the situations in which our proposal can be adapted.

Therefore, in the future, for robots to coexist with humans, it will be necessary to examine the relationship between appearance and interaction in terms of friendly speech and appropriate personality behaviors beyond limited situations. In recent years, small robots with only a face and torso have been used to perform front-desk duties in hotels. Regardless, the concept of the three-stage processing model proposed in this study can be used to design and verify such acceptable robots. Designing preferred robots based on human process flow will lead to the most acceptable robots and comfortable coexistence.

## Data Availability

The original contributions presented in the study are included in the article/Supplementary material, further inquiries can be directed to the corresponding authors.
